# Analyzing patient trust through the lens of hospitals managers—The other side of the coin

**DOI:** 10.1371/journal.pone.0250626

**Published:** 2021-04-26

**Authors:** Aviad Tur-Sinai, Royi Barnea, Orna Tal

**Affiliations:** 1 Department of Health Systems Management, The Max Stern Yezreel Valley College, Yezreel Valley, Israel; 2 School of Nursing, University of Rochester Medical Center, Rochester, NY, United States of America; 3 Assuta Health Services Research Institute, Tel Aviv, Israel; 4 Shamir Medical Center (Assaf Harofeh), Be’er Ya’akov, Israel; 5 Israeli Center for Emerging Technologies (ICET), Tel Aviv, Israel; 6 Department of Management, Bar Ilan University, Ramat Gan, Israel; University of Haifa, ISRAEL

## Abstract

Trust is an essential element in patient-physician relationships, yet trust is perceived differently among providers and customers exist. During January-February 2020 we examined the standpoints of medical managers and administrative directors from the private and public health hospitals on patient-physician trust, using a structured questionnaire. Thirty-six managers in public and private hospitals (24 from the public sector and 12 from the private sector) responded to the survey. Managers in the private sector rated trust higher in comparison to managers in the public sector, including trust related to patient satisfaction, professionalism and accountability. Managers from public hospitals gave higher scores to the need for patient education and shared responsibility prior to medical procedures. Administrative directors gave higher scores to various dimensions of trust and autonomy while medical managers gave higher scores to economic considerations. Trust is a fundamental component of the healthcare system and may be used to improve the provision and quality of care by analyzing standpoints and comparable continuous monitoring. Differences in position, education and training influence the perception of trust among managers in the health system. This survey may allow policy makers and opinion leaders to continue building and maintaining trust between patients and care providers.

## Introduction

Patient trust is a fundamental cornerstone in patient-physician encounters [1], yielding improvement in health outcomes, continuity of care and satisfaction [2, 3]. Although the definition of "trust" is still vague, it contains components of loyalty, personal care, consistent longitudinal care [4], regular good experiences, increasing knowledge [5], contentment [6], sometimes acknowledged as "personal doctoring" [7].

Although all partners involved in patient care; i physicians, patients and their families and medical managers, believe that trust has become one of the foundations of modern healthcare, these relationships are often unbalanced. Individuals who seek healthcare services may perceive the medical environment as precarious due to the risk of medical errors [8] Complete trust in the physician may be fragile, and requires to increase physicians’ awareness, compassion [9] and training [10]. Moreover, in the overloaded work environment of physicians, patient expectations are not always fully understood or accepted by the treating physician.

Within the medical world, surgical procedures emphasize the patient-physician relationship. The informed consent procedure prior to surgery is a unique opportunity to achieve patients’ trust by using medical knowledge in order to help patients overcome their fear [11], improve satisfaction and enhance patients’ experience.

Achieving patient trust in a hospital setting requires more than a simple person to person encounter. Patients’ experience is often associated with waiting times for hospital appointments, and threatening events such as a treatment or surgery. Sometimes the experience reflects the weakness of the individual patient by highlighting socioeconomic gaps in accessibility to care [12].

Lack of resources and poor leadership may impact the health system, described as "key factors leading to providers’ inadequate trust, contributed to poor quality services, driving a perverse cycle of negative patient–provider relations" [13]. A factor analysis of patient perception of trust showed that the contribution of empathy and assurance was relatively low and explained 8% and 5.6% of trust respectively, in comparison to comfortable facilities and appearance (21%), confidentiality (18.7%) and staff responsiveness (16%) [14]. The type of provider (public/private), hospital experience, the format of insurance coverage, freedom of choice also affect trust and distrust, alongside the configuration of the healthcare system [15].

A cross-sectional analysis in 23 countries [16] revealed that trust in physicians differs among health systems and may correlate to health strategy and policy, and to the nature of the health system itself. Trust in physicians was significantly higher in decommodified countries that highlight health as a basic human right (e.g., the United Kingdom, Japan, Norway and the Netherlands) than in commodified countries such as United States (3.8 vs. 3.4; P = 0.0035). The net support of family members, representing high "social trust" [17], can play a role in the physician-patient-family trust triangle.

The Israeli healthcare system is publicly funded, relying on governmental accountability. Provision of care is available through four health maintenance organizations (HMOs) to any citizen needing medical attention regardless of the ability to pay. Three tiers of coverage are provided: Tier 1 is universal coverage under the National Health Insurance Law (1995) to all residents in accordance with a standard positive list (“basket”) including surgery, acute and rehabilitative inpatient care, medications, and community care. For medications, tests, and treatments in the community, however, copays are charged. Tier 2 comprises supplemental insurance arrangements delivered by the HMOs, including a list of treatments, services, and medications, but only a few palliative medications, and no life-saving ones. Furthermore, the HMOs’ supplemental policies differ in terms, types and extent of oncological coverage. Tier 3 contains various private health insurance policies (personal and group). People who lack private coverage are, of course, susceptible to pay for care not included in the basic basket.

Due to dwindling resources in the Israeli public healthcare system [18], surgical waiting times may be prolonged, causing many patients to seek treatment in private healthcare. Although the proportion of surgical positions in private hospitals is only 11% of all surgery positions in Israel, approximately a quarter of all surgeries are conducted in the private healthcare system [19], where patients use their private (commercial) insurance or HMO’s supplementary insurance. Notably, only elective procedures are performed in private hospitals [20].

The researchers decided to regard patient-physician trust from two opposite directions: a "top-down" approach centered on the patient and excellency in care, and a "bottom-up" approach that involves medium-level managers, medical executives and heads of clinical departments in patient-physician dialogues. Although trust is currently well-established in patient-physician interactions, its vague definition may lead to differences in perceptions, standpoints and behavior among various stakeholders–service providers as well as customers/patients. To understand and improve the patient-physician dialogue, this study was aimed to understand health managers’ perceptions of trust. Specifically, (1) to examine the standpoints of the medical leadership (comprising leading clinical experts who stance as medical managers and administrative executives) on patient-physician trust; and (2) to identify trends and compare similarities and gaps between the private and public health sectors.

## Methods

This cross-sectional study was conducted during January-February 2020 among physicians in managerial positions (clinical and department managers and hospital medical executives) and administrative managers working in general hospitals in the public and private sectors in Israel. Altogether the 24 public and the 4 private general hospitals in the country were approached, thus reflecting leaders from the entire healthcare system.

### Sampling technique

37 managers were approached and 36 agreed to participate: 24 in public and 12 in the private hospitals in Israel. Potential participants were chosen by their involvement in policy decision-making discussion groups and relevance to the study’s aim. The proportion of participants from each sector was determined by the ratio of private to public general hospitals in Israel (5:11). All participants were approached personally with a request to participate in the study and all of them agreed to participate. None of the managers refused to participate in the survey.

Prior to administering the questionnaire, the purpose and procedure of the survey were explained in a telephone call.

### Ethical issues

The study was formally approved by the Ethical Committees of Assuta Medical Center and Shamir Medical Center. Each medical center’s Helsinki committees approved the study ethics, the procedure, and the survey questionnaire (reference numbers 0108-19-ASF and 0034-19-ASMC). The participants were informed in writing that their answers would be kept secret for the purposes of the study and they were required to declare their consent to this. All the participants were provided with information regarding the research purpose, confidentiality of information, and right to revoke the participation without prior justification.

### Questionnaire and data collection

A structured questionnaire was used to collect opinions via personal interviews. The questionnaire included 61 items in 4 parts, based on a grounded theory [21], with a Cronbach’s alpha of 0.9172: (A) Components of trust in caregivers and providers (3 questions), values and ideological principles, such as autonomy, satisfaction, accountability freedom of choice and economic implication of care utilization (8 questions); (B) Potential implications of surveys as tools to assess patients as customers (8 questions), personal values of the participating manager (4 questions); (C) Perceived understanding and trust of patients in ten selected operative procedures (30 questions), the effect of low patient trust on caregivers (one question); (D) participant demographics (7 questions). The participants rated each item on a scale of 1 (fully disagree) to 10 (fully agree).

The operative procedures discussed in Section C of the questionnaire were chosen by an expert committee to represent highly prevalent procedures (with significant activity in private and public hospitals) in a variety of clinical fields and populations. These included: adenoidectomy, appendectomy, cholecystectomy, hysterectomy, mastectomy, repair of inguinal hernia, repair of undiscerning testicle, rhinoplasty, total hip replacement and total knee replacement.

### Statistical analysis

For each of the questionnaire items, means and Standard Deviations were calculated in accordance with the various reference groups—type of provider (public sector or private) and position of manager/director (director or administrative). The mean and the S.D. of each of the various dimensions were calculated the same way, including, as stated, references to several questions for each.

To check statistical variance among the reference groups in regard to each the dimension and research question, an independent t-test was performed. Also, a 95% CI was calculated in order to examine the difference in the mean for each of the dimensions, in accordance with the various reference groups, as well as the Cohen’s d effect size.

## Results

A total of 36 managers participated in the study: 24 participants (17 men and 9 women) from 5 public hospitals, and 12 participants (9 men and 3 women) from 4 private hospitals. Nineteen participants were physicians in clinical managerial positions and 17 were clinicians in executive managerial positions that also had medical management training.

### Service provider (private vs. public)

Managers working in the private sector (PRsM) rated core variables related to patient satisfaction and aspects of trust in physicians, the hospital and the healthcare system as a whole, higher compared to the average rating of managers working in the public sector (PBsM) (8.61 vs. 7.89, p = 0.04). They also rated variables related to autonomy and economic considerations higher than PBsM (while despite having observed a difference, it is not possible to draw a statistically supported conclusion) (7.43 vs. 6.85, p = 0.40 and 7.59 vs. 6.38, p = 0.23, respectively) (Table 1). PRsM also gave higher ratings, compared to PBsM, to variables relating to professionalism and accountability, such as physician accountability for best practice (9.17 vs. 6.96, p = 0.01), the hospital’s accountability to supply good care (while despite having observed a difference, it is not possible to draw a statistically supported conclusion) (8.42 vs. 7.50, p = 0.21), the freedom to choose a specific surgeon (while despite having observed a difference, it is not possible to draw a statistically supported conclusion) (8.36 vs. 7.75, p = 0.48), and payment as a factor in care provision from the perspective of the caregiver and the hospital (while despite having observed a difference, it is not possible to draw a statistically supported conclusion) (7.89 vs. 6.25, p = 0.17; 7.30 vs. 5.89, p = 0.49, respectively) (S1 Appendix). PRsM also gave higher scores to the opportunity to use surveys as potential tools for assessing the patient as a customer. In contrast, PBsM gave higher ratings to two perceived beneficial implications of such a survey: being a tool for strategic planning (7.29 vs 6.69, p = 0.04), and the need to educate medical students to listen to their patients, and to integrate patients values and wishes in a shared decision making approach (while despite having observed a difference, it is not possible to draw a statistically supported conclusion) (9.28 vs 8.92 p = 0.23). PRsM gave higher scores to patients’ preliminary knowledge regarding the procedures (while despite having observed a difference, it is not possible to draw a statistically supported conclusion) (7.34 vs. 7.05, p = 0.49). On the other hand, PBsM gave higher scores to the need for patient’s education prior to medical procedures (while despite having observed a difference, it is not possible to draw a statistically supported conclusion) (7.95 vs. 7.49 p = 0.26) as well as to shared responsibility in decision making (8.70 vs. 7.28 p<0.01) (Table 1).

**Table 1:** Survey core variables by type of provider and role/position (mean).

Core variables		Type of provider				
Dimension	Parameter	Public sector N = 24	Private sector N = 12	P value	Diff, 95% CI	Effect size
Value	Trust	7.89 (1.05)	8.61 (0.77)	0.04	0.718 (0.024, 1.411)	0.78
	Autonomy	6.85 (2.05)	7.43 (1.66)	0.40	0.576 (-0.811, 1.963)	0.31
	Economic	6.38 (2.96)	7.59 (2.18)	0.23	1.216 (-0.817, 3.249)	0.47
The role of patient survey as a tool	As a planning tool	7.29 (1.70)	6.69 (2.40)	0.40	-0.602 (-2.037, 0.832)	0.29
	The patient as a player/ partner in policymaking	9.28 (0.78)	8.92 (0.93)	0.23	-0.361 (-0.959, 0.236)	0.42
Procedures	Patient preliminary knowledge	7.05 (0.95)	7.34 (1.59)	0.49	0.296 (-0.563, 1.154)	0.22
	Patient education/ explanation as a tool	7.95 (1.10)	7.49 (1.18)	0.26	-0.458 (-1.266, 0.349)	0.40
	Sharing responsibility/ partnership in care	8.70 (1.15)	7.28 (1.43)	<0.01	-1.413 (-2.308, -0.517)	1.09

Note. Parentheses denote standard deviation value.

An elaboration of the dimensions and a one-by-one examination of the ten specific operative procedures included in the survey revealed a consistent trend of higher average trust scores by PBsM compared to PRsM in eight of the ten operative procedures: appendectomy (9.00 vs. 6.55 respectively, p<0.01), gallbladder removal (8.63 vs, 7.18, p = 0.02), inguinal hernia (8.92 vs. 7.67, p = 0.01), hysterectomy (8.71 vs. 7.42, p = 0.01), mastectomy (9.08 vs. 8.36, p = 0.08) (an observed difference with proximity to p = 0.05 limit), rhinoplasty (8.38 vs. 7.42, p = 0.06) (an observed difference with proximity to p = 0.05 limit), undescended testicle repair (8.58 vs. 7.10, p = 0.04) and tonsillectomy (8.83 vs. 7.58, p = 0.01) (S2 Appendix).

Comparative scoring of the consequence of knowledge transfer from physicians to patients revealed dissimilarities among the participants; for example, PBsM rated the importance of providing knowledge or the added value of explanation/education significantly higher in hysterectomy (8.17 vs.7.00, p = 0.03) and tonsillectomy (8.50 vs 7.58 respectively, p = 0.06) (an observed difference with proximity to p = 0.05 limit) compared with PRsM (S2 Appendix). In other cases the opposite picture was revealed: PRsM rated the importance of providing knowledge on knee replacement and inguinal hernia significantly higher compared to PBsM (8.08 vs. 6.42, p = 0.01 and 9.00 vs. 7.46 p<0.01, respectively).

### The role and position of the participating managers (Medical Managers vs. Administrative Directors)

Administrative Directors (AD) had rated dimensions of trust and autonomy higher than medical managers (MM) (while despite having observed a difference, it is not possible to draw a statistically supported conclusion) (8.65 vs. 7.67, p<0.01 and 7.29 vs. 6.82, p = 0.47, respectively). AD also rated patients’ preliminary knowledge regarding the procedures and the need for patient education prior to medical procedures higher than MM (while despite having observed a difference, it is not possible to draw a statistically supported conclusion) (7.98 vs. 7.63, p = 0.36 and 7.29 vs. 7.01 p = 0.48, respectively). MM rated economic considerations and sharing responsibility regarding treatment decisions higher than AD (while despite having observed a difference, it is not possible to draw a statistically supported conclusion) (7.11 vs. 6.34, p = 0.42, and 8.41 vs. 8.02 p = 0.42, respectively) (Table 2).

**Table 2:** Survey core variables by type of provider and role/position (mean).

Core variables		Position of manager/director				
Dimension	Parameter	Medical N = 19	Administrative N = 17	P value	Diff, 95% CI	Effect size
Value	Trust	7.67 (0.93)	8.65 (0.86)	<0.01	0.975 (0.366, 1.582)	1.09
	Autonomy	6.82 (2.05)	7.29 (1.97)	0.47	0.469 (-0.844, 1.782)	0.23
	Economic	7.11 (2.62)	6.34 (2.95)	0.42	-0.762 (-2.679, 1.156)	0.28
The role of patient survey as a tool	As a planning tool	7.12 (1.89)	7.08 (2.03)	0.96	-0.035 (-1.387, 1.317)	0.02
	The patient as a player/ partner in policymaking	9.19 (0.82)	9.12 (0.88)	0.79	-0.075 (-0.651, 0.501)	0.08
Procedures	Patient preliminary knowledge	7.01 (1.39)	7.29 (0.92)	0.48	0.284 (-0.527, 1.094)	0.24
	Patient education/ explanation as a tool	7.63 (1.42)	7.98 (0.66)	0.36	0.351 (-0.417, 1.118)	0.32
	Sharing responsibility/ partnership in care	8.41 (1.46)	8.02 (1.34)	0.42	-0.382 (-1.337, 0.574)	0.28

Note: Parentheses denote standard deviation value.

Dimensions related to the perception of patient surveys as a managerial or planning tool or as a barometer to partnership in care showed even smaller difference between MM and AD (while despite having observed a difference, it is not possible to draw a statistically supported conclusion) (7.12 vs 7.08, p = 0.96 and 9.19 vs 9.12, p = 0.79, respectively).

MM rated trust within the scope of particular operative procedures consistently higher than AD, as demonstrated for appendectomy (8.79 vs. 7.56 respectively, p = 0.06) (an observed difference with proximity to p = 0.05 limit). In contrast, AD rated the importance of knowledge transfer to the patient or the added value of explanation/education significantly higher than MM in procedures such as mastectomy (8.19 vs. 7.26, p = 0.05), knee replacement (8.24 vs. 7.16, p = 0.02) and hip replacement (8.06 vs. 7.11, p = 0.04) (S2 Appendix).

A qualitative analysis of free text remarks added to the questionnaire revealed PRsM mentioned that the major forces leading to patient-physician trust are cohesiveness within the multidisciplinary team members, while PBsM emphasized professionalism as a leading vector. PRsM refere to economic incentives as obstacles to the perception of full trust; such as fair pricing, charges or mode of payment. In comparison, PBsM mentioned socioeconomic gaps or disparities in accessibility to care as barriers to full trust.

## Discussion

"Trust" is a complex entity, composed of sharing of knowledge, responsibility and satisfaction, and increases safety behavior and perception [22] when both sides rely on each other and have an incentive to join forces to keep a "contract". Traditionally surveys refer to either the patient’s standpoint or less frequently to physicians values, yet rarely introduce the perspective of the medical leadership. Our unique contribution focuses on the standpoints on medical managers, presenting differences according to their expertise, position and provider sector. The analysis revealed several trends, however we primarily focus on the most significant finding., The importance of trust as a general value was ranked higher by administrative managers compared to clinical managers and by managers in the private sector compared to managers in the public sector. On the contrary the value of patient- doctor partnership or "shared responsibility" was ranked higher by public sector managers in comparison with private sector managers, while position (medical vs administrative) showed no significant differences. A profound observation reveals administrative managers deeply appreciate trust among all stakeholders- the general practitioner, the surgeon and the entire healthcare. They also emphasized the caregiver’s responsibility as a key professional principle. Analysis by the type of provider showed private sector managers unsurprisingly highlighted the responsibility of the physician regarding the patient-doctor equation, moreover they suggest such surveys may be used as managerial tool for further planning and improvement.

Other topics investigated in our questionnaire revealed no significant differences among the groups of participants, although clues to trends were traced; for example PBsM gave ranked higher freedom of choice and considering patients’ preferences, alongside the added value of residents’ education and the opportunity of data sharing.

The same trend exists across almost all types of operative procedures examined, PRsM attributed greater importance of professionalism and accountability, probably due to the frequent attention drawn to these elements by their board of directors. This may be explained by the characteristics of the targeted encounter between the patient and the physician in the public health sector, shortly before the surgical procedure. In contrast, in the private healthcare system the patient-physician relationship starts in a consultation meeting prior to the surgery, and the patient has several opportunities to create trust with the care provider and to consider options for care before the operative procedure take place.

Our findings, which indicate higher trust levels among managers in the private health sector compared to the public setting are in line with Niv-Yagoda’s work, which showed an association between low levels of trust in the public healthcare system and the public’s perception regarding the importance of patient’s autonomy (e.g., selecting a surgeon) [23].

We believe that the executive managers rated trust higher than their counterparts due to their experience and holistic approach emphasizing the current focus on patient empowerment and the MoH strategic guidance to implement patient-centered policy. Interestingly, clinical professional experts highlighted economic issues, which are considered a barrier to consumption of health services, in particular, elective surgeries. Clinical managers may also consider the economic burden as a bigger threat to avoid maximal beneficial treatment to socially deprived populations.

The qualitative analysis of participants’ remarks added to the questionnaire, revealed differences in the major themes about factors influencing trust as well a spectrum of positive and negative sentiments: PRsM regard effective multidisciplinary teamwork as a leading force to trust, while economic barriers may reduce trust. PBsM believe professionalism is the foremost vector to gain trust, while socioeconomic gaps decrease trust.

As every Israeli resident is entitled by law to receive surgery in the public healthcare system, regardless of his/her financial resources, it is possible that PBsM expressed pointed to the importance of equity while PRsM highlighted economic incentives.

The sentiment in both sectors was positive "Trust exists and is a crucial element is healthcare, an essential need". Surprisingly PRsM described trust as a comprehensive, ongoing encounter, while PBsM referred to it as an acute episode or a "snapshot", explained by the episode of informed consent prior to surgery.

### Study limitations

Although we approached managers from various medical centers in the public and the private sector, the convenience sampling method may have limited our ability to generalize our findings, and the small sample may have influenced the statistical strength. Additionally, as the managers were approached personally, a social desirability may have affected their answers. Further research may clarify trends that are emerging yet non significantly, we suggest a to compare our findings with another health system.

## Conclusions

Trust is an essential component of healthcare systems and as such should be further nourished and maintained by both patients and care providers. By focusing on manager perspectives, we were able to provide a complementary view to that of patients, yet far from completion. This survey may allow policy makers and opinion leaders to continue building and maintaining trust between patients and care providers. Moreover, it would be interesting to explore whether the Covid-19 breakout that appeared after our survey was already completed, would have an influence on this fragile patient-physician relationship.

## Supporting information

S1 Appendix Overall perception of trust by service provider or type of expertise (mean). (DOCX)

S2 Appendix Perceived understanding and trust of patients in selected operative procedures (mean). (DOCX)
