# Supplementary material for: Analyzing patient trust through the lens of hospitals managers—The other side of the coin
Source: PLoS One. 2021 Apr 26;16(4):e0250626. doi: 10.1371/journal.pone.0250626 (PMC8075209; doi:10.1371/journal.pone.0250626)
Supplement: S1 Appendix — (DOCX) [file pone.0250626.s001.docx]

**S1 Appendix. Overall perception of trust by service provider or type of expertise (mean)**

|  |  |  | **Type of service provider** | | | **Type of expertise** | | |
| --- | --- | --- | --- | --- | --- | --- | --- | --- |
|  | **Question no.** | **Question** | **Private sector n=12** | **Public sector n=24** | **P value** | **Professional N=19** | **Administrative N=17** | **P value** |
| General trust | 1 | Do care recipients trust the Israeli healthcare system? | 7.25  (1.48) | 6.83  (1.76) | 0.49 | 6.42  (1.98) | 7.58  (0.94) | 0.03 |
| General trust | 2 | Do care recipients in Israel trust their personal caregiver when they undergo a procedure? | 8.67  (0.98) | 8.25  (1.39) | 0.36 | 8.05  (1.65) | 8.76  (0.44) | 0.09 |
| General trust | 3 | Do care recipients in Israel trust their direct caregivers (primary physician, family physician, etc.)? | 8.25  (1.71) | 7.21  (2.02) | 0.14 | 6.47  (2.06) | 8.76  (0.83) | <0.01 |
| Trust in caregiver | 4 | Are they satisfied with their caregivers? | 8.83  (1.40) | 8.46  (1.98) | 0.56 | 8.63  (1.83) | 8.53  (1.81) | 0.87 |
| Institution trust | 5 | Are they satisfied with the hospital where they received care? | 8.75  (1.14) | 8.92  (1.25) | 0.70 | 8.68  (1.34) | 9.06  (1.03) | 0.36 |
| Caregiver trust | 6 | Extent of trust in caregiver | 9.33  (0.78) | 8.33  (2.33) | 0.16 | 8.00  (2.47) | 9.41  (0.87) | 0.03 |
| Institution trust | 7 | Extent of trust in caregiving environment (hospital department) | 8.83  (1.47) | 8.58  (1.74) | 0.67 | 8.42  (1.77) | 8.94  (1.48) | 0.35 |
| Caregiver trust | 8 | Caregiver's responsibility to provide professional optimal care | 9.17  (0.94) | 6.96  (2.63) | 0.01 | 7.00  (2.60) | 8.47  (2.03) | 0.07 |
| Institution trust | 9 | Hospital's responsibility to assure optimal care | 8.42  (1.31) | 7.50  (2.27) | 0.21 | 7.37  (2.03) | 8.29  (1.96) | 0.17 |
| Autonomy | 10 | Care recipient's freedom to choose a specific caregiver | 8.36  (1.29) | 7.75  (2.69) | 0.48 | 7.89  (2.79) | 8.00  (1.75) | 0.90 |
| Autonomy | 11 | Care recipient's freedom to choose a specific hospital | 7.60  (3.44) | 8.29  (2.42) | 0.51 | 7.56  (3.11) | 8.69  (2.15) | 0.23 |
| Autonomy | 12 | To what extent can a survey among care recipients estimate the caregiver's independence in deciding on a specific procedure? | 7.10  (2.51) | 5.63  (3.13) | 0.20 | 5.79  (3.05) | 6.40  (3.02) | 0.56 |
| Autonomy | 13 | To what extent can a survey among care recipients reflect care recipients' independence in making decisions about their health in general? | 6.55  (3.01) | 5.75  (2.95) | 0.47 | 6.05  (2.79) | 5.94  (3.21) | 0.91 |
| Economic | 14 | To what extent do you think it appropriate to examine the question of funding in the context of trust in caregiver? | 7.89  (2.32) | 6.25  (3.22) | 0.17 | 6.94  (2.93) | 6.44  (3.27) | 0.64 |
| Economic | 15 | To what extent do you think it appropriate to examine the question of funding in the context of trust in hospital? | 7.30  (2.16) | 5.89  (3.34) | 0.49 | 7.17  (2.66) | 6.25  (3.41) | 0.39 |
| Implications of caregiver's stance for caregiver and policy (the survey as a planning tool) | 16 | To what extent can a survey among care recipients be helpful in planning staff efficiencies? | 5.60  (3.17) | 7.89  (1.92) | 0.01 | 7.31  (2.38) | 7.07  (2.79) | 0.78 |
| Implications of caregiver's stance for caregiver and policy (the survey as a planning tool) | 17 | To what extent can a survey among care recipients be helpful in preventing mistakes? | 7.18  (2.71) | 6.83  (2.59) | 0.85 | 7.42  (2.27) | 6.63  (2.94) | 0.37 |
| Implications of caregiver's stance for caregiver and policy (the survey as a planning tool) | 18 | To what extent can a survey among care recipients be helpful in information-sharing among caregivers? | 6.82  (2.48) | 7.38  (2.20) | 0.51 | 6.89  (2.26) | 7.56  (2.31) | 0.39 |
| Implications of caregiver's stance for caregiver and policy (the survey as a planning tool) | 19 | To what extent can a survey among care recipients be helpful in enhancing doctors' awareness of keeping information confidential if the topic comes up in the survey? | 6.82  (2.40) | 6.92  (2.50) | 0.91 | 6.84  (2.57) | 6.94  (2.35) | 0.91 |
| Care recipient as a player in the system/a partner in policymaking | 20 | How important is it to you to take the care-recipient's preferences into account? | 8.67  (1.37) | 8.96  (0.91) | 0.45 | 8.79  (0.98) | 8.94  (1.20) | 0.68 |
| Care recipient as a player in the system/a partner in policymaking | 21 | To what extent do you consider "listening to the care recipient," for example, valuable for medical students? | 9.25  (0.97) | 9.58  (0.78) | 0.27 | 9.47  (0.84) | 9.47  (0.87) | 0.99 |
| Care recipient as a player in the system/a partner in policymaking | 22 | To what extent do you consider "co-opting the care recipient in the decision-making process" a value? | 8.83  (1.03) | 9.29  (1.08) | 0.23 | 9.32  (1.11) | 8.94  (1.03) | 0.30 |
| "Mirror" question | 23 | To what extent do you believe that care recipients consider attitude (kindness, respect) an indicator of trust in the caregiver? | 9.50  (0.52) | 8.67  (1.69) | 0.11 | 8.84  (1.64) | 9.06  (1.25) | 0.66 |

Note. Parentheses denote standard deviation value
