# Supplementary material for: Analyzing patient trust through the lens of hospitals managers—The other side of the coin
Source: PLoS One. 2021 Apr 26;16(4):e0250626. doi: 10.1371/journal.pone.0250626 (PMC8075209; doi:10.1371/journal.pone.0250626)
Supplement: S2 Appendix — (DOCX) [file pone.0250626.s002.docx]

**S2 Appendix. Perceived understanding and trust of patients in selected operative procedures (mean)**

|  |  |  |  | Type of service provider | | | Type of expertise | | |
| --- | --- | --- | --- | --- | --- | --- | --- | --- | --- |
|  |  | Question no. | Question | Private sector N=12 | Public sector N=24 | P value | Professional N=19 | Administrative N=17 | P Value |
| Caregiver as consumer (knowledge) | Inguinal hernia surgery | 24 | To what extent, in your opinion, is the procedure conventional and common? | 9.00  (1.04) | 7.46  (1.56) | <0.01 | 7.89  (1.70) | 8.05  (1.48) | 0.76 |
| Explanation as a tool (sharing responsibility) |  | 25 | To what extent is the procedure focused and clear to the care recipient? | 8.25  (1.21) | 7.83  (1.63) | 0.44 | 8.05  (1.58) | 7.88  (1.45) | 0.74 |
| Trust |  | 26 | To what extent does a care recipient's discourse about the procedure reflect a dialogue of trust surrounding work familiar to you and the conditions under which the procedure is performed? | 7.67  (1.50) | 8.92  (1.18) | 0.01 | 8.84  (1.42) | 8.12  (1.32) | 0.12 |
| Caregiver as consumer (knowledge) | Appendectomy | 27 | To what extent, in your opinion, is the procedure conventional and common? | 8.17  (1.47) | 8.38  (1.13) | 0.64 | 8.42  (1.39) | 8.18  (1.07) | 0.56 |
| Explanation as a tool (sharing responsibility) |  | 28 | To what extent is the procedure defined, focused, and clear to the care recipient? | 7.83  (1.19) | 8.17  (1.40) | 0.49 | 8.16  (1.54) | 7.94  (1.09) | 0.63 |
| Trust |  | 29 | To what extent does a care recipient's discourse about the procedure reflect a dialogue of trust surrounding work familiar to you and the conditions under which the procedure is performed? | 6.55  (2.01) | 9.00  (1.25) | <0.01 | 8.79  (1.78) | 7.56  (1.86) | 0.06 |
| Caregiver as consumer (knowledge) | Knee replacement | 30 | To what extent, in your opinion, is the procedure conventional and common? | 8.08  (1.56) | 6.42  (1.59) | 0.01 | 6.68  (1.89) | 7.29  (1.57) | 0.30 |
| Explanation as a tool (sharing responsibility) |  | 31 | To what extent is the procedure defined, focused, and clear to the care recipient? | 8.00  (1.13) | 7.50  (1.53) | 0.32 | 7.16  (1.68) | 8.24  (0.75) | 0.02 |
| Trust |  | 32 | To what extent does a care recipient's discourse about the procedure reflect a dialogue of trust surrounding work familiar to you and the conditions under which the procedure is performed? | 8.42  (1.31) | 8.42  (1.28) | 1.00 | 8.47  (1.35) | 8.35  (1.22) | 0.78 |
| Caregiver as consumer (knowledge) | Hip replacement | 33 | To what extent, in your opinion, is the procedure conventional and common? | 7.58  (1.73) | 6.67  (1.69) | 0.14 | 6.74  (1.88) | 7.24  (1.56) | 0.40 |
| Explanation as a tool (sharing responsibility) |  | 34 | To what extent is the procedure defined, focused, and clear to the care recipient? | 7.83  (1.11) | 7.42  (1.50) | 0.40 | 7.11  (1.59) | 8.06  (0.90) | 0.04 |
| Trust |  | 35 | To what extent does a care recipient's discourse about the procedure reflect a dialogue of trust surrounding work familiar to you and the conditions under which the procedure is performed? | 8.17  (1.19) | 8.42  (1.44) | 0.61 | 8.37  (1.54) | 8.29  (1.16) | 0.87 |
| Caregiver as consumer (knowledge) | Tonsillectomy | 36 | To what extent, in your opinion, is the procedure conventional and common? | 7.83  (1.99) | 7.42  (1.64) | 0.51 | 7.47  (1.84) | 7.65  (1.69) | 0.77 |
| Explanation as a tool (sharing responsibility) |  | 37 | To what extent is the procedure defined, focused, and clear to the care recipient? | 7.58  (1.38) | 8.50  (1.29) | 0.06 | 8.26  (1.37) | 8.12  (1.41) | 0.76 |
| Trust |  | 38 | To what extent does a care recipient's discourse about the procedure reflect a dialogue of trust surrounding work familiar to you and the conditions under which the procedure is performed? | 7.58  (1.68) | 8.83  (1.17) | 0.01 | 8.53  (1.39) | 8.29  (1.57) | 0.64 |
| Caregiver as consumer (knowledge) | Rhinoplasty | 39 | To what extent, in your opinion, is the procedure conventional and common? | 6.33  (2.19) | 6.71  (1.71) | 0.58 | 7.00  (2.03) | 6.12  (1.58) | 0.16 |
| Explanation as a tool (sharing responsibility) |  | 40 | To what extent is the procedure defined, focused, and clear to the care recipient? | 7.08  (1.08) | 7.75  (1.45) | 0.17 | 7.58  (1.46) | 7.47  (1.28) | 0.82 |
| Trust |  | 41 | To what extent does a care recipient's discourse about the procedure reflect a dialogue of trust surrounding work familiar to you and the conditions under which the procedure is performed? | 7.42  (1.56) | 8.38  (1.35) | 0.06 | 8.11  (1.73) | 8.00  (1.17) | 0.83 |
| Caregiver as consumer (knowledge) | Hysterectomy | 42 | To what extent, in your opinion, is the procedure conventional and common? | 7.00  (2.00) | 6.54  (1.41) | 0.44 | 6.56  (1.46) | 6.82  (1.78) | 0.63 |
| Explanation as a tool (sharing responsibility) |  | 43 | To what extent is the procedure defined, focused, and clear to the care recipient? | 7.00  (1.48) | 8.17  (1.40) | 0.03 | 7.68  (1.89) | 7.88  (0.99) | 0.70 |
| Trust |  | 44 | To what extent does a care recipient's discourse about the procedure reflect a dialogue of trust surrounding work familiar to you and the conditions under which the procedure is performed? | 7.42  (1.51) | 8.71  (1.20) | 0.01 | 8.47  (1.68) | 8.06  (1.09) | 0.39 |
| Caregiver as consumer (knowledge) | Mastectomy | 45 | To what extent, in your opinion, is the procedure conventional and common? | 8.09  (1.58) | 7.50  (1.29) | 0.25 | 7.26  (1.28) | 8.19  (1.38) | 0.05 |
| Explanation as a tool (sharing responsibility) |  | 46 | To what extent is the procedure defined, focused, and clear to the care recipient? | 7.83  (1.27) | 8.42  (1.21) | 0.19 | 8.05  (1.43) | 8.41  (1.00) | 0.40 |
| Trust |  | 47 | To what extent does a care recipient's discourse about the procedure reflect a dialogue of trust surrounding work familiar to you and the conditions under which the procedure is performed? | 8.36  (1.03) | 9.08  (1.10) | 0.08 | 8.95  (1.22) | 8.75  (1.00) | 0.61 |
| Caregiver as consumer (knowledge) | Undescended testicle | 48 | To what extent, in your opinion, is the procedure conventional and common? | 6.09  (2.81) | 5.83  (1.99) | 0.76 | 5.72  (2.16) | 6.12  (2.37) | 0.61 |
| Explanation as a tool (sharing responsibility) |  | 49 | To what extent is the procedure defined, focused, and clear to the care recipient? | 6.91  (2.02) | 7.71  (1.73) | 0.24 | 7.28  (2.14) | 7.65  (1.50) | 0.56 |
| Trust |  | 50 | To what extent does a care recipient's discourse about the procedure reflect a dialogue of trust surrounding work familiar to you and the conditions under which the procedure is performed? | 7.10  (2.13) | 8.58  (1.69) | 0.04 | 8.17  (2.15) | 8.13  (1.71) | 0.95 |
| Caregiver as consumer (knowledge) | Gallbladder removal | 51 | To what extent, in your opinion, is the procedure conventional and common? | 7.64  (1.75) | 7.54  (1.38) | 0.86 | 7.39  (1.61) | 7.76  (1.35) | 0.46 |
| Explanation as a tool (sharing responsibility) |  | 52 | To what extent is the procedure defined, focused, and clear to the care recipient? | 7.82  (0.98) | 8.04  (1.40) | 0.64 | 7.78  (1.48) | 8.18  (1.01) | 0.36 |
| Trust |  | 53 | To what extent does a care recipient's discourse about the procedure reflect a dialogue of trust surrounding work familiar to you and the conditions under which the procedure is performed? | 7.18  (1.89) | 8.63  (1.50) | 0.02 | 8.22  (1.86) | 8.12  (1.65) | 0.86 |

Note. Parentheses denote standard deviation value
